# Comparative Study of Safety and Efficacy of Angiotensin-Receptor Blockers and Anti Amyloid-ß Monoclonal Antibodies for the Treatment of Alzheimer’s Disease: A Systematic Review

**DOI:** 10.7759/cureus.43984

**Published:** 2023-08-23

**Authors:** Kamran Shahid, Yonas Tamene, Shefali P Mody, Kaiser O Sadiq, Yogamba M Shivakumar, Eshwar Burra, Shivana Ramphall

**Affiliations:** 1 Internal Medicine/Family Medicine, California Institute of Behavioral Neurosciences & Psychology, Fairfield, USA; 2 Internal Medicine, California Institute of Behavioral Neurosciences & Psychology, Fairfield, USA; 3 Medicine, California Institute of Behavioral Neurosciences & Psychology, Fairfield, USA; 4 General Surgery, California Institute of Behavioral Neurosciences & Psychology, Fairfield, USA

**Keywords:** anti-amyloid therapy, alzheimer's dementia, monoclonal antibodies, angiotensin-receptor blockers, alzheimer's disease

## Abstract

Amyloid-ß (Aß) plaques and Neurofibrillary tangles are hallmarks of Alzheimer's disease (AD) pathology. Recent advances to find a cure for AD have led to the exploration of Anti-Aß monoclonal antibodies and angiotensin-receptor blockers (ARBs). The antibodies can decrease plaque formation or remove already formed plaques. ARBs increase angiotensin II (AT2) levels and decrease the effect of AT2 on the AT1 receptor (AT1R). This systematic analysis reviews evidence of monoclonal antibodies (Aducanumab, Lecanemab, Donanemab, and Solanezumab) and ARBs in managing AD. An in-depth methodical search was conducted across PubMed, Science Direct, and Mendeley. PRISMA 2020 guidelines were followed for this study. Randomized control trials for antibodies and ARBs and one retrospective cohort study were included. The comparison was made among studies that shared similar measured outcomes. Antibodies were found to be more effective than ARBs, with Aducanumab and Lecanemab being the most effective. ARBs, on the other hand, were found to be the safer choice. Further trials of longer duration and larger sample sizes are needed to explore both groups' long-term safety and efficacy.

## Introduction and background

Alzheimer's disease (AD) is the leading cause of dementia, accounting for 60 to 80 percent of all dementia cases worldwide [1]. It is an age-related disease with an incidence rate of 1% at 65 years and can increase to 8% by 85 years of age [2]. AD can present clinically with a wide spectrum of signs and symptoms. Memory impairments, especially with newly developed memories and recalling events. Impairments in focus lead to difficulty with planning, dealing with numbers, and following conversations. Disorientation in time and place can lead to difficulty with the timeline of events and getting lost in once-familiar locations [3].

Amyloid-ß (Aß) plaques and Neurofibrillary tangles (NFTs) are the pathological hallmarks of AD. Aß plaques are extracellular accumulations formed due to abnormally folded Aß, which are formed due to the catabolism of Amyloid precursor protein (APP). NFTs are an intracellular accumulation of hyperphosphorylated tau protein [4]. Acute inflammation on its own plays a protective role by removing Aß plaques, but the persistence of inflammation decreases the plaque-removing capability of microglia, resulting in increased plaque deposition. Plaque deposition in cholinergic neurons leads to their degradation, giving rise to a cholinergic deficit which, in turn, alters the blood-brain barrier, making it more permeable, further disrupting the removal of Aß plaques, and allowing faulty passage of metabolites [5].

While the catabolism of APP is vital for neuronal protection, inappropriate cleavage by ß and γ- secretase can produce Aß40 and Aß42. While both Aß peptides can accumulate, Aß42 has a higher tendency to form plaques and can stimulate pro-inflammatory cytokines such as Tumor Necrosis factor-α and increase oxidative stress [6]. Based on these findings, passive immunization using anti-Aß antibodies has been a subject of interest in searching for a cure for AD. Animal studies have shown promising results in terms of improvements in both cognition and amyloid burden, further increasing the appeal of this strategy [7].

Hypertension is potentially a modifiable risk factor for AD, as recent studies have shown hypertension plays a significant role in its pathogenesis [8]. Angiotensin-converting enzyme (ACE) is responsible for converting angiotensin I (AT1) to angiotensin II (AT2), which plays a role in hypertension [9] and has been directly implicated in the pathogenesis of Alzheimer's disease. AT2 increases oxidative stress, causes neuro-inflammation, and exhibits anticholinergic properties, all of which, as mentioned above, leads to decreased clearance of Aß plaques [10]. AT2, after its production, can take part in 4 different pathways [11]: a) ACE1/AT2/AT1 receptor (AT1R): This pathway is responsible for the role that AT2 plays in AD. This route gives rise to neuroinflammation, oxidative stress, increased blood-brain barrier permeability, decreased cerebral blood flow, and increased formation and deposition of Aß plaques; b) ACE1/AT2/AT2 receptor (AT2R): This neuroprotective and neuroregenerative pathway improves synaptic plasticity and increases cerebral blood flow; c) ACE2/AT1-7/Mas-receptor (MasR): The activation of MasR has a neuroprotective effect and plays a part in decreasing Aß plaques and NFTs; d) AT4/AT4 receptor (AT4R): Once thought to be a degradation product of AT2, AT4 binding to AT4R plays a positive role in memory and cognition by increasing cerebral blood flow and modulating synaptic plasticity. 

These findings and work on animal models [12-15] have increased interest in using Angiotensin receptor blockers (ARBs) to treat and manage Alzheimer's disease.

Considering the prevalence of AD, it is necessary to figure out how to effectively lessen its incidence and slow down the progression in the affected population. This systematic review aims to compare angiotensin receptor blockers and anti-Aß amyloid antibodies on their level of safety and effectiveness in human subjects. While on the one hand, ARBs have been around for decades and can easily and swiftly be repurposed and used, Anti-Aß antibodies are relatively new, and the long-term complications and possible interactions are not well understood. Ultimately, this review aims to advance the understanding of each treatment strategy's differential benefits and drawbacks.

## Review

Methodology

The Preferred Reporting Items for Systematic Reviews and Meta-Analyses (PRISMA) criteria conducted a methodical literature search. Full-text publications, paid and free, indexed in PubMed, ScienceDirect, and Mendeley were searched from inception to 2023, using the keywords "Alzheimer's Disease", "Monoclonal Antibody," and "Angiotensin Receptor Blocker". Table 1 provides the comprehensive search technique using the three data sources, and Table 2 shows the MESH strategy used for PubMed.

**Table 1:** Databases used and the search strategy using keywords

S.No	Database	Keyword	Search Result
1	Pubmed	Alzheimer's disease AND monoclonal antibody	2782
2	Pubmed	Alzheimer’s disease AND Angiotensin receptor blocker	197
3	ScienceDirect	Alzheimer's disease AND monoclonal antibody	581
4	ScienceDirect	Alzheimer’s disease AND Angiotensin receptor blocker	39
5	Mendeley	Alzheimer's disease AND monoclonal antibody	2139
6	Mendeley	Alzheimer’s disease AND Angiotensin receptor blocker	207

**Table 2:** Mesh strategy for PubMed

Mesh Database
Concept 1: Alzheimer’s disease: ( "Alzheimer Disease/drug therapy"[Majr] OR "Alzheimer Disease/prevention and control"[Majr] OR "Alzheimer Disease/therapy"[Majr] )
Concept 2: Angiotensin receptor Blockers: ( "Angiotensin Receptor Antagonists/therapeutic use"[Majr] OR "Angiotensin Receptor Antagonists/toxicity"[Majr] )
Concept 3: Monoclonal Antibody: ( "Antibodies, Monoclonal/adverse effects"[Majr] OR "Antibodies, Monoclonal/pharmacology"[Majr] OR "Antibodies, Monoclonal/therapeutic use"[Majr] )
Full Mesh
(( "Alzheimer Disease/drug therapy"[Majr] OR "Alzheimer Disease/prevention and control"[Majr] OR "Alzheimer Disease/therapy"[Majr] )) AND ( "Angiotensin Receptor Antagonists/therapeutic use"[Majr] OR "Angiotensin Receptor Antagonists/toxicity"[Majr] ) ((( "Alzheimer Disease/drug therapy"[Majr] OR "Alzheimer Disease/prevention and control"[Majr] OR "Alzheimer Disease/therapy"[Majr] ))) AND ( "Antibodies, Monoclonal/adverse effects"[Majr] OR "Antibodies, Monoclonal/pharmacology"[Majr] OR "Antibodies, Monoclonal/therapeutic use"[Majr]

After the search completion, duplicates were found and removed by two reviewers, and the relevant publications were chosen by inspecting the titles and abstracts. Articles in English from 2013 to 2023 were included. Randomized control trials and Cohort studies were included, and the age range of the population was 50-90 years. Other types of dementia, e.g., vascular dementia, were excluded, along with studies done on animal subjects.

Study Selection

We looked for studies that assessed for efficacy and/or safety of anti-Aß antibodies and angiotensin receptor blockers for treating AD. Randomized control trials and cohort studies were included. We excluded all studies that were not available in the English language, animal studies, gray literature, case reports, book chapters, editorials, and systematic reviews. Articles were first screened using the titles and abstracts only, and relevant articles were later screened using the full text.

Data Extraction and Analysis

We extracted data based on authors, year, study design, sample size, age (years), gender, intervention, efficacy, and safety. Two reviewers extracted and cross-checked data, and any disputes were solved. A narrative synthesis was performed on the extracted data.

Risk of Bias Assessment

Risk of bias (ROB) assessment was done using the Revised Cochrane risk-of-bias tool for randomized trials (RoB 2). The domains included in ROB were risk of bias arising from the randomization process, risk of bias due to deviations from the intended interventions, risk of bias due to missing outcome data, risk of bias in the measurement of the outcome, and risk of bias In the selection of the reported result. The traffic light plot was created using Risk-of-bias VISualization (robvis) tool [16].

Quality Appraisal for Cohort Study

We included one cohort study in this systematic review, and it was assessed using the Newcastle-Ottawa Quality Assessment Form for Cohort Studies, which has three domains (selection, Comparability, and Outcome) with a maximum score of eight points.

Results

Search Results

**Figure 1:**
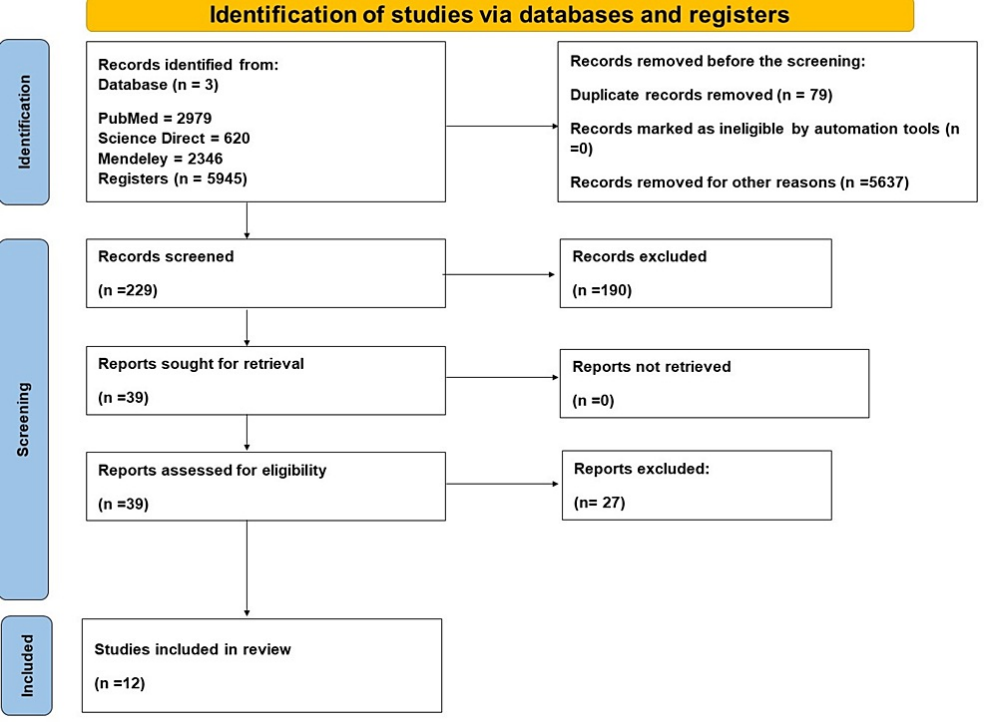
Prisma Flow chart of the literature and the search strategy

A thorough search from three databases yielded 5,945 articles, 79 duplicates were removed, and 5,637 articles were removed because they did not meet the inclusion criteria. The remaining 229 articles were screened using titles and abstracts, of which 39 remaining articles were assessed for eligibility, out of which only 12 were included in this review. Figure 1 shows the literature's PRISMA flowchart and the studies' search strategy [17].

ROB of Clinical Trials

**Figure 2:**
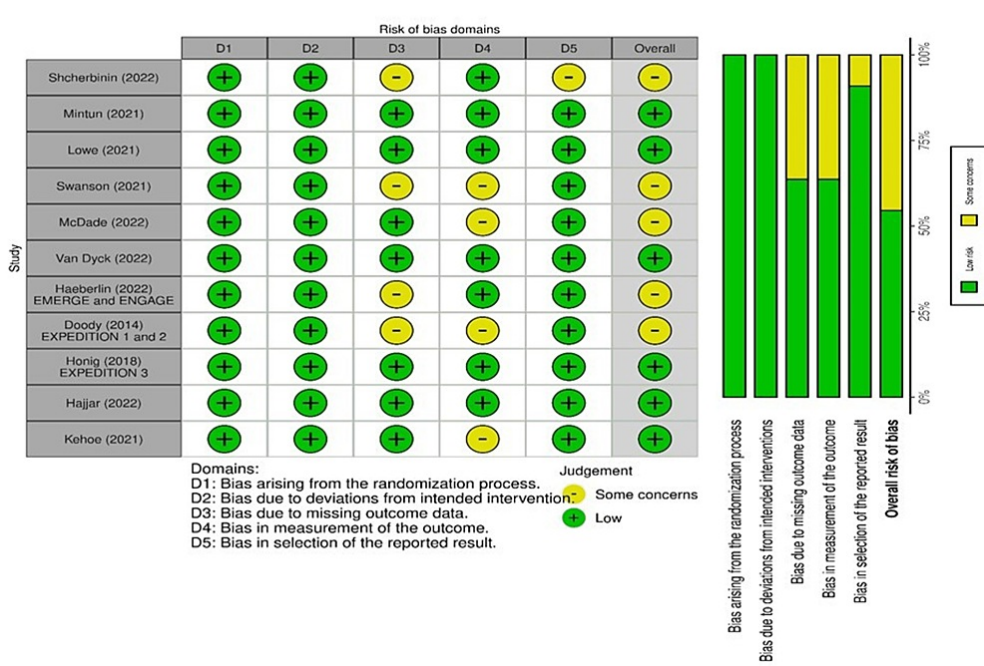
ROB traffic light plot and summary plot Lowe (2021) [18]; Mintun (2021) [19]; Shcherbinin (2022) [20]; Swanson (2021) [21]; Van Dyck (2023) [22]; McDade (2022) [23]; Doody EXPEDITION 1(2014) [24]; Doody EXPEDITION 2(2014) [24]; Honig Expedition 3 (2018) [25]; Haeberlein (2022) [26]; Kehoe (2021) [10]; Hajjar (2022) [27]

All included RCTs were either good or fair in quality. Graphical analysis using the traffic light and summary plot is shown in Figure 2.

Newcastle-Ottawa Quality Assessment

The included cohort study scored a score of seven out of the maximum eight points. Individual scores in each domain are shown in Table 3.

**Table 3:** NCOS for Cohort Studies. A study can be awarded a maximum of one point for each category except for the comparability of cohorts based on design or analysis.

Categories	Lee (2023) [28]
Representativeness of exposed cohort	1
Selection of the non-exposed cohort	1
Ascertainment of exposure	0
Demonstration that outcome of interest was not present at the start of the study	1
Comparability of cohorts on the basis of design or analysis(2 points)	2
Assessment of outcome	0
Was follow-up long enough for the outcome to occur	1
Adequacy of follow-up of the cohort	1
Total	7
Quality	GOOD

Characteristics of Included Studies

All included studies were published between 2014-2023. The following data were extracted from each article: Author, study design, sample size, age, gender, intervention, and follow-up period. A summary of the characteristics of included studies as well as descriptions of intervention, is described in Table 4.

**Table 4:** Characteristics of selected studies. IV= Intravenous; Q2W= every 2 weeks; Q4W= every 4 weeks; BACEi= ß-site amyloid precursor protein cleaving enzyme inhibitor; OLE= Open-label extension; RAS= renin-angiotensin system; M= Male; F= Female.

Author (Year)	Design	Sample Size	Age	Gender	Intervention	Follow-Up period
Lowe (2021) [18]	Triple Blinded, Randomized, placebo-controlled, six-arm parallel, single and multiple dose Phase One b studies	Total= 61 Donanemab= 46 Placebo=15	54 to 90	M=27 F=34	Cohort 1-3: Single IV dose of Donanemab of 10mg/kg, 20mg/kg, 40mg/kg OR Placebo Cohort 4: Multiple IV doses of Donanemab 10mg/kg OR placebo Q2W for 24 weeks Cohort 5-6: Multiple IV doses of Donanemab 10mg/kg, 20mg/kg OR placebo Q4W for 72 weeks	Cohort 1 and 2: 72 weeks Cohort 3: 24 weeks Cohort 4: 48 weeks Cohort 5 and 6: 12 weeks
Mintun (2021) [19]	Double-blind, Randomized, placebo-controlled Phase two trial	Total=272; Donanemab= 131; Placebo=126 15 participants, placed in a third group with a combination of BACEi and Donanemab, were omitted from the final result	60 to 85	M=127 F=145	Donanemab: IV administration of 700 mg for first 3 doses and then 1400mg for doses afterward. Placebo: IV administration. Dosage frequency: Q4W for up to 72 weeks.	76 weeks
Shcherbinin (2022) [20]	Double-Blind, Randomized, placebo-controlled Phase two trial	Total= 272; Donanemab = 131; placebo= 126; 15 participants placed on a combination of BACEi and Donanemab were omitted from the final analysis	60 to 85	M=127 F=145	Donanemab Q4W: 700mg for the first 3 doses and 1400 mg afterward for up to 72 weeks with blinded dose reduction evaluations at 24 and 52 weeks.	48 weeks
Swanson (2021) [21]	Double-blind, Randomized, Placebo-controlled, Bayesian design phase two b trial	Total= 854 Lecanemab=609 Placebo=245	50 to 90	M=445 F=409	Lecanemab: Arm 1:2.5mg/kg biweekly; Arm 2:5mg/kg monthly Arm 3:5mg/kg biweekly; Arm 4:10mg/kg monthly Arm 5:10mg/kg biweekly; Placebo: biweekly	18 months
Van Dyck (2023) [22]	Double-blind, Randomized, Placebo-controlled, Parallel group phase three trial	Total= 1734; Lecanemab= 859 Placebo=875	50 to 90	M=827 F=907	IV Lecanemab 10mg/kg biweekly OR Placebo biweekly	18 months
McDade (2022) [23]	Double-blind, Randomized, Placebo-controlled, Parallel Group, Bayesian design trial	Core= Same as Swanson (2021) OLE: Prior Core placebo = 42 Prior core Lecanemab 10mg/kg biweekly= 37 10mg/kg Lecanemab biweekly=180	50 to 90	Core=same as Swanson (2021) OLE: M=133 F=126	Core: Same as Swanson (2021); OLE: 10 mg/kg biweekly	Core: 3 months OLE: up to 24 months
Doody, EXPEDITION 1(2014) [24]	Double-Blind, Randomized, phase three trial	Total=1012; Solanezumab= 506; Placebo= 506	≥55	M=426 F=586	Solanezumab 400mg IV Q4W for 18 months OR placebo	80 weeks
Doody, EXPEDITION 2(2014) [24]	Double-Blind, Randomized, phase three trial	Total= 1040; Solanezumab= 521; Placebo= 519	≥55	M=471 F=569	Solanezumab 400mg IV Q4W for 18 months OR placebo	80 weeks
Honig, Expedition 3 (2018) [25]	Double-blind, Randomized, placebo-controlled, Phase three trial	Total= 2129; Solanezumab =1057; Placebo=1072	55-90	M=898 F=1231	Solanezumab 400mg IV Q4W for 76 weeks or placebo	80 weeks
Haeberlein (2022) [26]	EMERGE and ENGAGE were both double-blind, Randomized, Placebo-controlled, Phase three trials	EMERGE: Total=1643; Placebo= 549, 1 not dosed Aducanumab 3 or 6 mg/kg= 547, 4 not dosed. Aducanumab 10mg/kg= 547 Total dosed=1638 ENGAGE: Total=1653; Placebo= 548, 3 not dosed Aducanumab 3 or 6 mg/kg= 549, 2 not dosed Aducanumab 10mg/kg= 556, 1 not dosed Total dosed=1647	50-85	EMERGE: M=798 F=840 ENGAGE: M=784 F=863	Aducanumab: low dose (3 or 6 mg/kg target dose), high dose (10 mg/kg target dose), or placebo IV infusion every 4 weeks for up to 76 weeks	78 weeks
Kehoe (2021) [10]	Double-blind, randomized, placebo-controlled, Phase two trial	Total=211; Losartan= 105; Placebo= 106	≥55	M=127 F=84	Oral 100mg Losartan or placebo for 12 months	12 months
Hajjar (2022) [27]	Double-blind, Randomized, Placebo-controlled trial	Total= 77 Candesartan= 38 Placebo= 39	≥50	M=29 F=48	Candesartan= oral 8mg daily with dose increments every 2 weeks until the maximum dose (32 mg) is reached or patients experience side effects. Placebo= matched daily oral	12 months
Lee (2023) [28]	Population-based, retrospective cohort study	Total= 57,420; RAS users= 28,710 Non-RAS users= 28,710	≥60	M=26,205 F=31,215		July 1^st^, 2009 to December 31st 2019

Discussion 

Mechanism of Action

**Figure 3:**
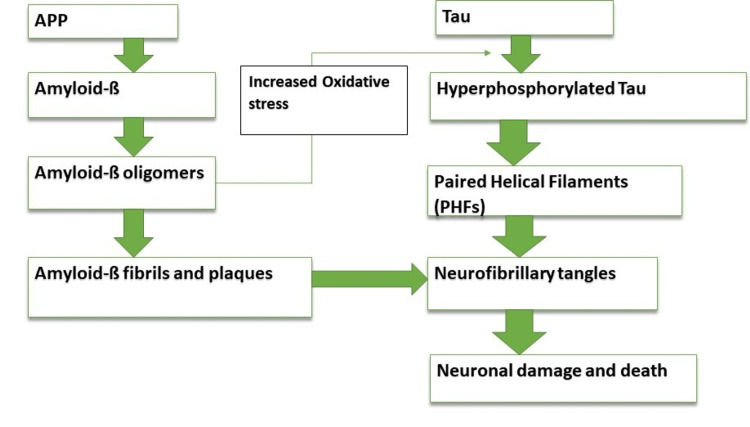
Simplified representation of the amyloid cascade. APP= Amyloid precursor protein Image credits: Dr. Kamran Shahid

Anti-Aß Monoclonal Antibodies: Monoclonal antibodies act on different stages of the amyloid cascade, as shown in Figure 3. The main focus of this systematic analysis will be on four monoclonal antibodies: Aducanumab, Lecanemab, Donanemab, and Solanezumab, as these have the most data available.

Aducanumab in a human IgG1 monoclonal antibody binds to oligomeric and fibrillar aggregates of Aß and increases clearance [1]. Donanemab is an IgG1 antibody that targets the N-terminal pyroglutamate Aβ epitope [29], reducing the amyloid burden by clearing existing plaques [18]. Lecanemab, an IgG1 antibody that binds to oligomeric and protofibrillar Aß aggregates, thereby preventing the formation of new plaques and increasing the reduction of pre-existing plaques (21). Lastly, Solanezumab, an IgG1 antibody, helps in Alzheimer's disease (AD) by increasing the clearance of soluble Aß [25].

**Figure 4:**
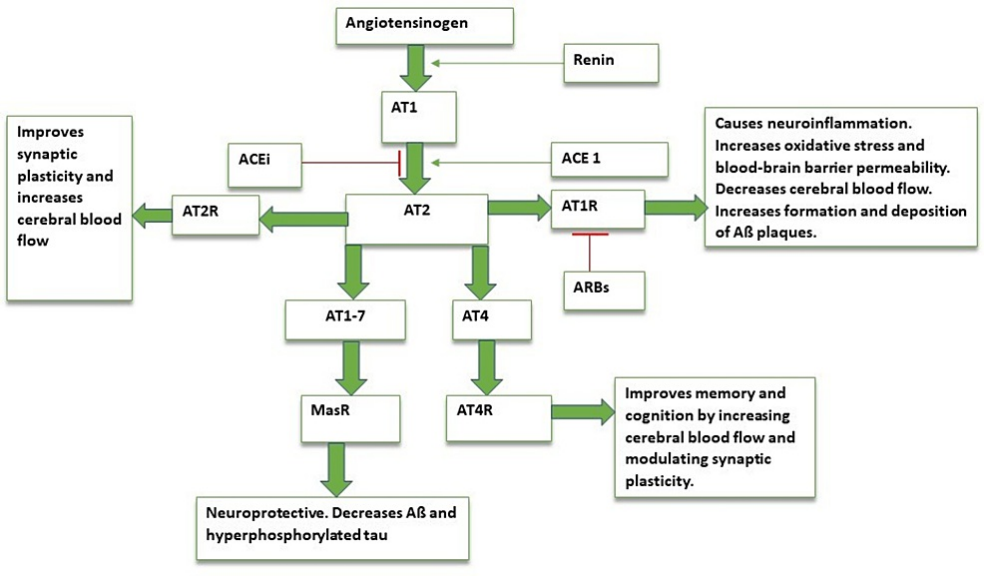
Simplified role of Angiotensin in AD. AT1= angiotensin 1; AT2= angiotensin II; ACEi= Angiotensin converting enzyme inhibitor; ACE 1= Angiotensin converting enzyme 1; AT1R= Angiotensin 1 receptor; ARBs= Angiotensin receptor blockers; AT2R= Angiotensin 2 receptor; AT4R= Angiotensin 4 receptor; AT4= Angiotensin 4; AT1-7= angiotensin 1-7; MasR= Mas receptor. Image credits: Dr. Kamran Shahid

Angiotensin-Receptor Blockers: Angiotensin-converting enzyme inhibitors (ACEi) act by inhibiting the conversion of AT1 to AT2 and, in doing so, decreases the formation and deposition of plaques via the ACE1/AT2/AT1R pathway. A decrease in AT2 also inhibits its beneficial role in the brain. On the other hand, angiotensin-receptor blockers (ARBs) act further down in the cascade by blocking the binding of AT2 to angiotensin I receptor (AT1R). This, unlike ACEis, only inhibits the pathway that promotes the pathogenesis of AD. The increase in AT2 levels caused by ARBs diverts AT2 to the pathways that exhibit beneficial effects on cognition, as summarized in Figure 4. ARBs also upregulate neprilysin, transthyretin, and insulin-degrading enzyme, which facilitate the clearance of Aß peptides [30].

Efficacy

Understanding the Outcomes: Cognition was assessed using multiple scoring criteria demonstrating disease progression. The assessments used were the Global Clinical Dementia Rating-Sum of Boxes (CDR-SB), Alzheimer's Disease Assessment Scale-Cognitive Subscale (ADAS-Cog), Alzheimer's disease composite score (ADCOMS), Mini-mental state exam (MMSE), and Alzheimer's Disease Cooperative Study-Activities of Daily Living Scale for use in Mild Cognitive Impairment (ADCS-MCI-ADL). A summary of the different assessments, along with the method of data interpretation, is given in Table 5. For the sake of comparison, only those cognitive assessments used in most of the articles were included in this review.

**Table 5:** Cognition assessments used and their interpretation CDR-SB= Global Clinical Dementia Rating-Sum of boxes; ADAS-Cog= Alzheimer's Disease Assessment Scale-Cognitive Subscale; ADCOMS= Alzheimer’s disease composite score; MMSE=Mini-mental state exam; ADCS-MCI-ADL=Alzheimer's Disease Cooperative Study – Activities of Daily Living Scale for use in Mild Cognitive Impairment

Name	Score range	Interpretation
Global Clinical Dementia Rating-Sum of boxes (CDR-SB)	0 to 18	A higher score indicates greater impairment
Alzheimer's Disease Assessment Scale-Cognitive Subscale (ADAS-Cog) 13/14	ADAS-Cog 13: 0 to 85 ADAS-Cog 14: 0 to 90	A higher score indicates greater impairment
Alzheimer’s disease composite score (ADCOMS)	0 to 1.97	A higher score indicates greater impairments
Mini-mental state exam (MMSE)	0 to 30	A lower score indicates greater impairment
Alzheimer's Disease Cooperative Study – Activities of Daily Living Scale for use in Mild Cognitive Impairment (ADCS-MCI-ADL)	0 to 53	A lower score indicates greater impairment

Amyloid positron emission tomography (PET) was used to measure amyloid burden. The results were obtained in Centiloid (CL) with higher values indicating higher load.

Biomarkers assessed were cerebrospinal fluid (CSF) and/or plasma concentrations of Aß40, Aß42 or an Aß42/Aß40 ratio, with higher values indicating better clearance [31].

Anti-Aß monoclonal antibodies: The trailblazer-ALZ [20] focused on amyloid reduction with Donanemab treatment. The study showed a mean difference (MD) between Donanemab and placebo of -0.14 for pTau217, signifying a greater decrease in tau load in the Donanemab group and MD of 0.01 for Aß40/42 ratio, showing a greater rise in Donanemab group. A total of 46 participants (40%) achieved complete amyloid clearance in the Donanemab group.

Trail done by Mintun et al. [19] showed an MD of -0.36 in CDR-SB and -1.86 in ADAS-Cog13, showing better cognition in the Donanemab group. MD of MMSE was 0.64, signifying an improvement in the Donanemab group, but the authors deemed these findings statistically insignificant. Eighty-nine participants (67.8%) in the Donanemab group achieved amyloid-negative status by week 76. At week 76, the MD in the amyloid burden on amyloid PET was -85.06 CL, showing that Donanemab was effective in amyloid clearance. Overall, this trial showed that Donanemab moderately improved cognition and removed plaques compared to placebo.

Lowe et al. [18] were designed to have six cohorts of participants all receiving different dosages. In the first three cohorts, there was an MD of -16.5 CL, -40.0 CL, and -49.6 CL in amyloid PET for a single dose of 10mg/kg, 20mg/kg, and 40mg/kg respectively. In the multi-dose cohorts (cohort 4-6) the MD in reduction of Amyloid were -55.8 CL in 10mg/kg Q2W, -50.2 CL in 10mg/kg Q4W, and -58.4 CL in 20mg/kg Q4W. Both single and multi-dose cohorts showed a swift reduction in brain amyloid with a total of 11 participants (24%) achieving complete amyloid clearance.

Swanson et al. [21] designed to have five treatment arms with different Lecanemab dosages. The 2.5mg/kg monthly and 5mg/kg monthly and biweekly showed no difference when compared to placebo. The 10mg/kg biweekly and monthly showed statistically significant improvements compared to placebo with 10mg/kg biweekly being identified as the effective dose 90%(ED90). Change from baseline at 18 months for ADCOMS showed an MD of -0.043 from placebo for 10mg/kg biweekly, -0.240 for CDR-SB, and -2.052 for ADAS-Cog14, using ANCOVA analysis with control multiple imputation procedure. For ED90 PET standardized uptake value ratio (SUVR) showed an MD -60.1 CL. Even though the primary endpoint of reduction in ADCOMS was not achieved, Lecanemab showed dose-dependent improvement in participants as compared to placebo. 

A trial conducted by McDade et al. [23] was a continuation of the two b trial to include a gap period of 24 months (range 9-59) and then an open-label phase where participants received a dose of 10mg/kg biweekly for up to 24 months. The gap phase showed that the beneficial effects of Lecanemab persisted throughout the off-drug period. During the OLE phase, the study showed MD of -0.66, -0.10, -0.66, and 38.75CL for CDR-SB, ADCOMS, ADAS-Cog14, and amyloid burden (CL) respectively. Lecanemab showed beneficial effects throughout both the treatment and gap phase with an increase in exposure leading to significant improvements. 

The phase three trial on Lecanemab by van Dyck et al. [22] was done with Lecanemab 10mg/kg biweekly or placebo. The results were an MD from baseline at 18 months of -0.050 for ADCOMS, -0.45 for CDR-SB, -1.44 for ADAS-Cog14, 2.0 for ADCS-MCI-ADL and -59.12CL for the amyloid burden on PET, showing that Lecanemab reduced amyloid levels and decelerated rate of clinical decline in participants with early AD.

EMERGE and ENGAGE [26] were two phase three trials done on Aducanumab. ENGAGE was unable to meet both primary and secondary endpoints and results were deemed statically insignificant by the authors. EMERGE on the other hand showed that a higher dose (10mg/kg) was better than both placebo and low dose (3 or 6mg/kg) with MD from placebo of -0.39 for CDR-SB, -1.40 for ADAS-Cog13, 0.6 for MMSE and 1.7 for ADCS-ADL-MCI showing a dose-dependent reduction in AD pathogenesis.

EXPEDITION 1 and EXPEDITION 2 [24] were phase three trials for Solanezumab. In EXPEDITION 1, the MD from baseline at the end of the follow-up period was 0.1 for CDR-SB, -1.4 for ADAS-Cog14, 0.6 for MMSE, -0.4 for ADCS-ADL but changes of MMSE and CDR-SB were deemed statistically insignificant by authors. EXPEDITION 1 also measured total CSF Aß40 and Aß42 levels with MD from placebo of 3227.6pg/ml and 713.7pg/ml respectively at the end of the trial. In EXPEDITION 2, the MD from baseline at the end of follow-up was -0.3 for CDR-SB, -1.6 for ADAS-cog14, 0.8 for MMSE, and 1.6 for ADCS-ADL with the result for CDR-SB deemed statistically insignificant by authors. This trial also measured total CSF Aß40 and Aß42 levels with MD from placebo at the end of the trial of 3033.1pg/ml and 402.8pg/ml respectively. Solanezumab underperformed in both trials with no significant improvements in cognition. 

EXPEDITION 3 [25] was conducted for further investigations into the efficacy of Solanezumab. The MD at the end of the trial was -0.34 for CDR-SB (deemed statistically insignificant by authors), -0.8 for ADAS-Cog14, 0.49 for MMSE, and 1.35 for ADCS-ADL. EXPEDITION 3 failed to show the benefit of Solanezumab on the primary outcome (ADAS-Cog14) and the treatment effect was smaller compared to the trials previously done.

Angiotensin-receptor blockers: Hajjar et al. [27] conducted a trial on the effect of Candesartan on non-hypertensive patients. The trial showed an increase in levels of Aß40 and Aß42 with MD to placebo at 12 months being 1211.95 pg/ml and 49.51 pg/ml respectively. The trial showed no statistically significant difference in values of CDR-SB but cognition was improved on Trail making test (TMT) part B and TMT part B-A. This trial showed that Candesartan increased the clearance of plaques and had positive effects on cognition.

Kahoe et al. [10] conducted a phase two clinical trial for Losartan. The trial found no significant effect of Losartan on brain atrophy or cognition with MD at the end of the trial of -0.52 for ADAS-Cog and -0.33 for MMSE.

**Figure 5:**
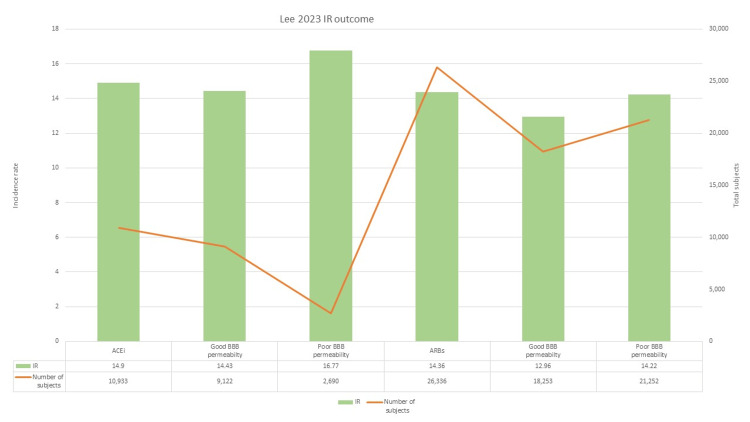
Incidence rate outcomes BBB= Blood brain barrier; IR= Incidence rate; ACEi= Angiotensin converting enzyme inhibitors; ARBs= Angiotensin receptor blockers

Lee et al. [28] conducted a cohort study to evaluate the effect of RAS-inhibiting drugs on AD. The trial also took into account the degree of BBB permeability of individual drugs. ACEi in general had an IR of 14.90 and ARBS had an IR of 14.36. ACEi and ARBs with higher BBB permeability showed a lower IR of 14.43 and 12.96 respectively as compared to those with lower BBB permeability (ACEi=16.77, ARBs=14.22). Overall, this cohort study showed ARBs were far better at lowering the incidence of AD and that BBB permeability is an important factor to consider when prescribing. A graphical representation of outcomes is shown in Figure 5.

Safety

Anti-Aß Monoclonal Antibodies: The main safety outcomes included in this review were Amyloid related imaging abnormalities (ARIA) and serious adverse events (defined as adverse events that result in death, require hospitalization, are life-threatening, or result in a disability/incapacity). ARIA can be further divided into ARIA-E, cerebral edema, and ARIA-H, including superficial siderosis and cerebral micro-hemorrhages. ARIA is a well-known and common complication of monoclonal antibody treatment. Studies by Shcherbinin et al. [20] and Mcdade et al. [23] were not included in the safety assessment as they did not have safety outcome measures. A breakdown of safety outcomes is given in Figure 10.

**Figure 6:**
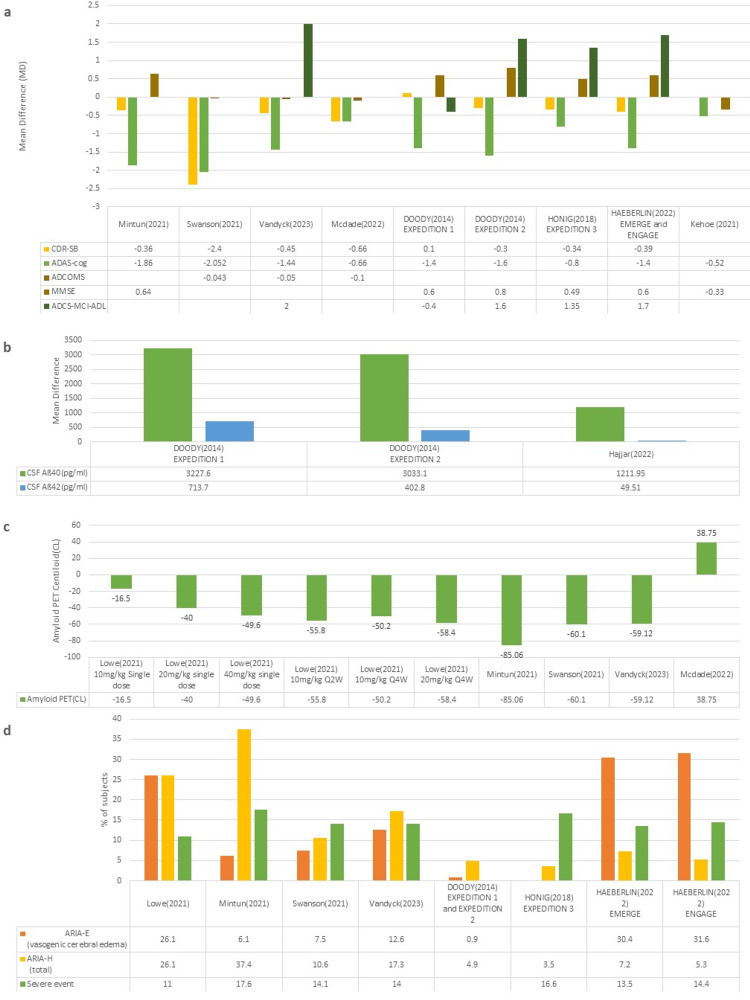
Graphical analysis of data for safety and efficacy a: Cognition measures outcomes; b: Biomarker changes; c: Changes in amyloid PET; d: Anti-Aß monoclonal antibodies safety CDR-SB=Global Clinical Dementia Rating-Sum of boxes; ADAS-Cog=Alzheimer's Disease Assessment Scale-Cognitive Subscale; ADCOMS= Alzheimer’s disease composite score; MMSE= Mini-mental state exam; ADCS-MCI-ADL=Alzheimer's Disease Cooperative Study – Activities of Daily Living Scale for use in Mild Cognitive Impairment; CSF= Cerebrospinal fluid; PET= positron emission tomography; CL=Centiloid; ARIA=Amyloid related imaging abnormalities; ARIA-E= Vasogenic cerebral edema; ARIA-H= Superficial siderosis and cerebral micro-hemorrhages

Angiotensin-Receptor Blockers: Hajjar et al. [27] found Candesartan to be well tolerated with only five participants (four in Candesartan and one in placebo) experiencing symptomatic hypotension (defined as sitting BP <100/40 taken five minutes apart). A total of 23 adverse events were reported in the candesartan group compared to 22 in the placebo group. Kahoe et al. [10] showed Losartan to be well tolerated at intervention doses, with only 22 reported adverse events in the treatment group and 20 in the placebo group. Overall, both trials on ARBs showed that they were well tolerated in both hypertensive and normotensive participants. A graphical representation of data for safety and efficacy is shown in Figure 6a-6d.

Limitations

One of the main limitations of this review was a lack of completed RCT data available for ARBs which can exaggerate the comparison between the groups. A cohort study added little value to the comparison because it did not compare changes in biomarkers or cognition endpoints shared by the other trials. Additionally, each study used different variables for biomarkers and cognition, which made the comparison difficult, which is why trials were compared with each other if they shared similar outcomes. Lastly, some articles used MMSE, an unreliable test in the early stages of AD [32].

## Conclusions

AD is a progressive, age-related disorder caused by damage and death of neuronal cells, leading to memory loss, behavioral changes, and cognitive decline. The main pathology behind AD is an accumulation of Aß plaques and NFTs. Monoclonal antibodies are a group of medications that act at various stages of the amyloid cascade and thus improve AD symptoms by either slowing the formation of new plaques or removing already formed ones. ARBs, especially those with high BBB penetrance, have been shown to have a neuroprotective effect by altering the route AT2 takes after formation. Monoclonal antibodies have shown promising results in efficacy, but their side effect profile makes them slightly unfavorable. ARBs as a group have been around for decades and have been studied extensively for possible side effects, but up till now, the evidence for their efficacy in AD is still under question. Among the Aß antibodies, Lecanemab and Aducanumab showed the highest efficacy in slowing clinical progression. Lecanemab had persistent benefits even through the gap phase, while Donanemab excelled at decreasing the amyloid burden. Solanezumab was the least efficacious of the group, with all three trials showing no clinical improvements. Among the ARBs, those with high BBB penetrance performed better. Compared to groups, Aß antibodies were far superior to ARBS in efficacy, but ARBs were much safer. More trials, preferably with a larger sample size, are needed to provide valuable insight into both drug groups' long-term safety and efficacy.
